# A phase 3 trial assessing the efficacy and safety of grass allergy immunotherapy tablet in subjects with grass pollen-induced allergic rhinitis with or without conjunctivitis, with or without asthma

**DOI:** 10.1186/1477-5751-12-10

**Published:** 2013-06-01

**Authors:** Kevin Murphy, Sandra Gawchik, David Bernstein, Jens Andersen, Martin Rud Pedersen

**Affiliations:** 1 Boys Town National Research Hospital, Boys Town, NE, USA; 2 Asthma and Allergy Associates, Upland, PA, USA; 3 Bernstein Clinical Research Center and University of Cincinnati College of Medicine, Cincinnati, OH, USA; 4 ALK-Abelló, Hørsholm, Denmark

**Keywords:** Allergic rhinitis, Allergic conjunctivitis, Allergic rhinoconjunctivitis, Allergy immunotherapy tablet, Randomized controlled trial, Specific immunotherapy, Sublingual immunotherapy

## Abstract

**Background:**

Design and execution of immunotherapy trials for seasonal allergies may be complicated by numerous factors including variable allergy testing methods, pollen levels, and timing and intensity of other seasonal allergens. We evaluated grass allergy immunotherapy tablet (AIT) treatment in North American adults with grass pollen-induced allergic rhinitis with or without conjunctivitis (AR/C), with/without asthma.

**Methods:**

Subjects age 18–65 with clinical history of grass pollen–induced AR/C, with/without asthma were randomized 1:1 to once-daily 2800 BAU Timothy grass AIT (oral lyophilisate, *Phleum pratense*, 75,000 SQ-T, containing approximately 15 μg of Phl p 5) or placebo. The AR/C symptom and medication scores were recorded daily. The primary end point was the average AR/C daily symptom score (DSS) during the entire grass pollen season (GPS). Ranked key secondary end points were Rhinoconjunctivitis Quality of Life Questionnaire (RQLQ) score, daily medication score (DMS), and percentage of well days, all over entire GPS. Safety was monitored through adverse event reporting.

**Results:**

Efficacy analysis included 289 subjects. Over the entire GPS, mean DSS was 6% lower with AIT versus placebo (5.69 vs. 6.06), but this difference was not statistically significant (p = 0.3475) despite significantly higher immunological response in the grass AIT group. No significant between-group differences were seen for key secondary end points. In general, DSS was high before GPS began and no clear relationship between DSS and grass pollen counts was seen during GPS. In post hoc analysis of subjects with pre-seasonal DSS ≤3, mean DSS and DMS were both significantly lower with grass AIT versus placebo (27%; p = 0.0327 and 68%; p = 0.0060, respectively). In this subgroup a relationship between DSS and grass pollen counts was observed. Grass AIT was generally well tolerated, with no events of anaphylactic shock or respiratory compromise.

**Conclusions:**

In this trial, 2800 BAU grass AIT did not demonstrate significant symptom improvement versus placebo. Lack of relationship between pollen count and symptom score in the study population, and post hoc findings among subjects with low pre-seasonal symptoms, suggest that the symptoms reported in this study were not primarily reflective of the effects of grass pollen exposure.

**Trial registration:**

NCT00421655

## Background

In Europe, Timothy grass allergy immunotherapy tablet (AIT) treatment is an approved means of administering immunotherapy sublingually to patients who are sensitized to Timothy and related grass pollens, and it has been approved by regulatory authorities for the disease-modifying treatment of grass pollen-induced allergic rhinitis with or without conjunctivitis (AR/C) [1]. Unlike symptomatic treatment, grass AIT has been shown to provide significant improvements in AR/C symptoms and medication use 2 years after cessation of treatment [1]. Conventional subcutaneous immunotherapy also has disease-modifying potential and has been used for more than a century, but it requires repeated in-office injections [2] and in some cases has been associated with severe anaphylaxis, including fatal reactions [3,4]. Sublingual allergy immunotherapy delivered via rapidly dissolving tablets is a relatively new, more convenient treatment modality that appears to be associated with a positive safety profile [5].

Compared with trials for symptomatic AR/C treatments, the design and execution of immunotherapy clinical trials is complex. In pharmacotherapy studies, subjects with AR/C are typically enrolled once their symptoms have reached a predefined level of severity, and the ability of the target agent to reduce symptoms can be observed within hours to days of initiation [6]. In trials of immunotherapy for seasonal allergies, treatment is initiated weeks or months prior to the onset of pollen season and the associated symptoms, to allow the treatment to modulate the immune system before the season starts [7]. Therefore, subjects are enrolled based on symptoms experienced in previous seasons; however, the severity of symptoms in previous seasons may not be an accurate predictor of upcoming symptoms due to several complicating factors [6]. Variable pollen levels, potential exposure to other allergens, pollutant exposure, weather patterns, allergen avoidance measures, disease progression, and methods of allergy testing can all exert effects on the results observed in a trial of seasonal allergy immunotherapy. We evaluated treatment with SCH 697243/MK-7243, a Timothy grass AIT formulation of 2800 bioequivalent allergen units (BAU), in North American adults with grass pollen-induced AR/C with or without asthma.

## Results and discussion

### Demographics and baseline characteristics

Of the 405 subjects who were screened, 329 subjects were randomized and were included in the safety analysis (76 [19%] subjects were screening failures). The efficacy analysis included 150 placebo-treated subjects and 139 grass AIT–treated subjects who completed at least 1 diary entry during the grass pollen season (GPS). In total, 140 (84%) and 136 (83%) subjects in the placebo and grass AIT groups completed the trial, whereas 26 (16%) and 27 (17%) in each group, respectively, withdrew prematurely. Among withdrawals, 15 (5%) were due to adverse events (AEs). The pattern of withdrawal was similar between treatment groups (Table 1).

**Table 1:** Subject disposition

	**Placebo**	**Grass AIT**
	**N (%)**	**N (%)**
Full analysis set (FAS)	166 (100%)	163 (100%)
Per protocol (PP)	119 (72%)	121 (74%)
Subjects with diary data (entire GPS)	150 (90%)	139 (85%)
Subjects with diary data (peak GPS)	143 (86%)	137 (84%)
Withdrawn from trial	26 (16%)	27 (17%)
Reason for withdrawal		
Withdrawal of consent	7 (4%)	8 (5%)
Lost to follow-up	5 (3%)	2 (1%)
Non-compliance with protocol	3 (2%)	1 (<1%)
Pregnancy	2 (1%)	0 (0%)
Adverse event	5 (3%)	10 (6%)
Other	4 (2%)	6 (4%)
Withdrawal initiated by		
Investigator	6 (4%)	7 (4%)
Sponsor	1 (<1%)	0 (0%)
Subject	19 (11%)	20 (12%)
Completed	140 (84%)	136 (83%)

9 of the subjects with data in the grass pollen season dropped out before the peak grass pollen season and thus did not provide any data in the peak grass pollen season.

% = percentage of the full analysis set (all randomized subjects). *AIT* Allergy immunotherapy tablet, *GPS* Grass pollen season.

Demographic and baseline characteristics were well balanced between treatment arms (Table 2). The majority of subjects were white (81%), mean age was 35.9 years, and mean duration of grass pollen allergy was 21 years. Asthma as a coexisting condition was well represented in both groups (grass AIT, 28%; placebo, 26%). Prevalence of sensitization to other allergens was high in both treatment groups. Pre-season symptom scores (over the 14 days before start of GPS) were high in both groups; 67% of subjects had a pre-season AR/C daily symptom score (DSS) >3.

**Table 2:** Baseline characteristics

	**Placebo (n = 166)**	**Grass AIT (n = 163)**
Female, no. (%)	88 (53%)	88 (54%)
Race, no. (%)		
White	134 (81%)	134 (82%)
Black	21 (13%)	21 (13%)
Hispanic/Latino	6 (4%)	4 (2%)
Non-smoker	121 (73%)	128 (79%)
Previous smoker, no. (%)	24 (14%)	26 (16%)
Smoker, no. (%)	21 (13%)	9 (6%)
Age, y		
Mean (SD)	35.9 (11.7)	35.9 (11.7)
Range	18-62	18-65
Subjects with asthma, no. (%)	43 (26%)	46 (28%)
Sensitive to non-grass allergens per skin prick, no. (%)		
White oak	74 (45%)	75 (46%)
White birch	84 (51%)	87 (53%)
Animal hair/dander (cat)	77 (46%)	79 (48%)
House dust mite	86 (52%)	93 (57%)
Mean duration of pre-treatment (weeks), [range]	16.2 [5.9, 23.9]	16.3 [6.4, 23.7]

*AIT *Allergy immunotherapy tablet, *DSS *Daily symptom score, *SD* Standard deviation.

### Grass pollen season

The GPS had a mean duration of 43 days. Mean daily pollen counts were 44 grains/m^3^ and 61 grains/m^3^ over the entire and peak GPS, respectively. The pre-seasonal treatment period was approximately 16 weeks (range: 6–24 weeks).

### Efficacy: primary and key secondary end points

**Figure 1:**
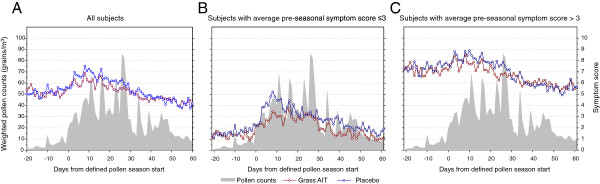
**Average daily symptom scores in (A) all subjects, (B) subjects with average pre-seasonal symptom score **≤ **3, and (C) subjects with average pre-seasonal symptom score >3. **AIT = allergy immunotherapy tablet.

In general, DSS was high both before and during the GPS, and for the entire study population no clear relationship between DSS and grass pollen counts was observed during the GPS (Figure 1A). Over the entire GPS, mean DSS was 6% lower in the grass AIT group compared with the placebo group (5.69 vs. 6.06), but this difference was not statistically significant (p = 0.3475) (Table 3). Despite high symptom scores both before and during the pollen season, symptomatic medication use was low in both groups, with 42% of grass AIT subjects and 43% of placebo subjects not using any rescue medication during the GPS. The mean daily medication score (DMS) was numerically lower (27%; p = 0.0827) in the grass AIT group (1.07) relative to placebo (1.47). No significant differences between groups were seen for average Rhinoconjunctivitis Quality of Life Questionnaire with standardized activities (RQLQ[S]) score (grass AIT = 1.36, placebo = 1.44; p = 0.5293) or for percentage of AR/C well days (grass AIT = 27%, placebo = 26%; p = 0.6965).

**Table 3:** DSS, DMS, RQLQ(S) scores* and percent well days during the grass pollen season

	**Placebo (n = 166)**	**Grass AIT (n = 163)**	**Difference**	**95% CI for difference**	***P *value**
DSS, mean (SE)	6.06 (.40)	5.69 (.39)	-0.37	[-0.41; 1.16]	0.3475
DMS, mean (SE)	1.47 (.22)	1.07 (.20)	-0.40	[-0.05; 0.85]	0.0827
RQLQ(S)† score, mean (SE)	1.44 (.12)	1.36 (.12)	-0.08	[-0.16; 0.32]	0.5293
Percent well days, mean (SE)	26.03 (3.13)	27.44 (3.29)	1.42	[-8.56; 5.73]	0.6965

*Scores were adjusted using the analysis of variance (ANOVA) model, with treatment group as fixed effect and pollen region as random effect. †RQLQ(S): number of observations: placebo = 840, Grass AIT = 801. *AIT* Allergy immunotherapy tablet, *CI *Confidence interval, *DMS *Daily medication score, *DSS *Daily symptom score, *RQLQ(S) *Rhinoconjunctivitis quality of life questionnaire with standardized activities, *SE *Standard error.

### Other efficacy end points

**Figure 2:**
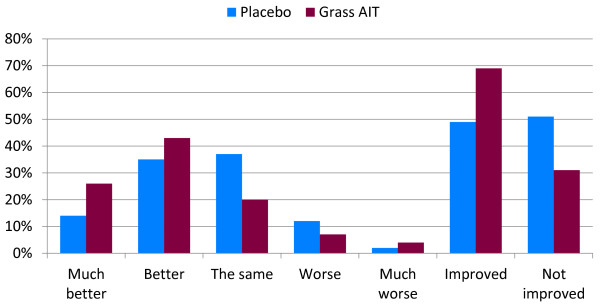
**Summary of overall global evaluation. **Overall assessment of pollen season compared to previous season. AIT = allergy immunotherapy tablet.

At the end of the GPS, the subjects answered the question, “Compared to your rhinoconjunctivitis symptoms in the previous grass pollen season, how have you felt overall in this grass pollen season?” Scoring is summarized in Figure 2. Data were pooled into the binary end point of improved (including those who answered “much better” or “better”) or not improved (those who answered “the same,” “worse,” or “much worse”). Results demonstrate that 69% of the subjects in the grass AIT group indicated improvement, compared with 49% who received placebo (odds ratio 2.24, p = 0.0010).

Additional efficacy variables (listed in Methods) failed to show significant differences between grass AIT and placebo.

### Post hoc subgroup analysis of efficacy

A post hoc analysis divided subjects into those with low pre-seasonal symptoms (DSS ≤3; 33% of all subjects) and those with high pre-seasonal symptoms (DSS >3; 67% of all subjects). In those with pre-seasonal DSS ≤3, mean DSS and mean DMS were both significantly lower over the GPS in the grass AIT group compared with the placebo group (27%; p = 0.0327 and 68%; p = 0.0060 respectively). Furthermore, the symptom scores in this subgroup closely corresponded to pollen exposure (Figure 1B). In subjects with pre-season DSS >3, no significant differences between grass AIT and placebo were seen for mean DSS or DMS (p > 0.05), and no clear relationship between DSS and grass pollen counts was observed (Figure 1C).

Although a clinical history of a potentially overlapping seasonal or perennial allergic disease was an exclusion criterion for this study, post hoc efficacy analyses were performed on subject subsets based on sensitization profiles to assess the effect of non-grass allergens on efficacy outcomes. These subsets included subjects who were SPT-negative to 1) oak, 2) birch, 3) house dust mite (HDM), and 4) hair and dander. An additional subset included only subjects who were monosensitized to grass pollen. Results in these subsets were consistent with those seen in the entire study population, in that no significant difference between grass AIT and placebo was observed in any of these subsets for the outcomes of mean changes in DSS or DMS.

### Immunologic measures

**Figure 3:**
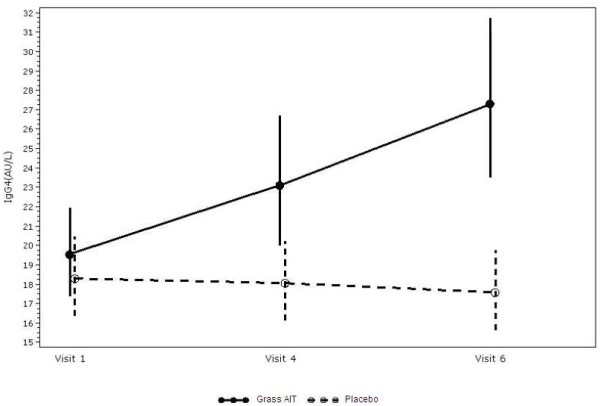
**Specific IgG4 over time. **AIT = allergy immunotherapy tablet.

Specific IgE and IgG4 levels were similar in both groups at baseline (visit 1; screening). Between visit 1 and visit 4 (pre-season), specific IgE levels increased in the grass AIT group, indicating an immunologic response to treatment. In the placebo group, specific IgE levels increased only after the start of the GPS and remained significantly lower compared with the grass AIT group. By the time of the pre-season visit, change from baseline in log-transformed IgG4 levels were significantly greater in the grass AIT group compared with those in the placebo group (p < 0.0001) (Figure 3). This treatment effect continued through the end of the season (p < 0.0001). A significantly higher induction of IgE-blocking antibodies as compared to baseline was observed for the grass AIT group than for the placebo group (p <0.0001) at both the pre-season visit and the end-of-season visit.

### Safety

Grass AIT treatment was generally well tolerated. There were no events of anaphylactic shock or respiratory compromise. No new safety signals were detected. Table 4 shows AEs reported after the beginning of treatment; treatment-emergent AEs were experienced by 121/163 subjects (74%) in the grass AIT group and 101/166 subjects (61%) in the placebo group. Discontinuations due to AEs were infrequent with both grass AIT treatment (10/163; 6%) and placebo (5/166; 3%). Six of the 10 withdrawals in the grass AIT group were considered to be possibly or probably treatment-related.

**Table 4:** Summary of treatment-emergent AEs

	**Placebo (n = 166)**		**Grass AIT (n = 163)**	
	N (%)*	**Events**	N (%)*	**Events**
All	101 (61)	258	121 (74)	454
Causality				
Probably related	11 (7)	23	73 (45)	184
Possibly related	13 (8)	29	20 (12)	29
Unlikely related	93 (56)	206	95 (58)	241
Severity				
Mild	70 (42)	146	100 (61)	268
Moderate	57 (34)	94	75 (46)	156
Severe	14 (8)	18	20 (12)	30

*Represents all subjects experiencing AE of each causality/severity; each subject could experience AEs in multiple categories of causality/severity. *AE* Adverse event, *AIT *Allergy immunotherapy tablet.

Treatment-related AEs were experienced by 57% of subjects in the grass AIT group and 15% of subjects in the placebo group. Table 5 lists the treatment-related AEs with an incidence of 5% or greater. The most common treatment-related AEs reported in the grass AIT group were ear pruritus, mouth edema, oral pruritus, oral paresthesia, and throat irritation. The median number of consecutive days in which local application-site reactions were reported to occur at any time during the day in the grass AIT group ranged from 1 to 16 days. Most treatment-related AEs were mild to moderate in severity both in the grass AIT group (percentage of subjects experiencing mild AEs: 61%; moderate, 46%; severe: 12%) and in the placebo group (mild: 42%; moderate, 34%; severe: 8%) (Table 4). Eight severe treatment-related AEs occurred in 6 subjects. Severe treatment-related AEs reported in the grass AIT group included 2 episodes each of urticaria and diarrhea, and 1 episode each of Eustachian tube obstruction, abdominal pain, and lip blister. There was also 1 severe treatment-related headache in the placebo group.

**Table 5:** Adverse events experienced by 5% or more of all treated subjects

	**Treatment-emergent AEs**		**Treatment-related AEs**	
**AEs, no. (% of subjects experiencing)**	**Placebo (n = 166)**	**Grass AIT (n = 163)**	**Placebo (n = 166)**	**Grass AIT (n = 163)**
Ear pruritus	1 (<1)	16 (10)	1 (<1)	16 (10)
Mouth edema	0 (0)	9 (6)	0 (0)	9 (6)
Oral pruritus	1 (<1)	29 (18)	1 (<1)	28 (17)
Nasopharyngitis	24 (14)	23 (14)	0 (0)	1 (<1)
Sinusitis	6 (4)	12 (7)	1 (<1)	0 (0)
URTI	15 (9)	17 (10)	0 (0)	0 (0)
Headache	12 (7)	8 (5)	1 (<1)	1 (<1)
Paraesthesia oral	2 (1)	14 (9)	2 (1)	14 (9)
Throat irritation	4 (2)	24 (15)	4 (2)	23 (14)
Urticaria	0 (0)	8 (5)	0 (0)	6 (4)

*AE *Adverse event, *AIT *Allergy immunotherapy tablet, *URTI* Upper respiratory tract infection.

Two treatment-related asthma events were reported during the treatment period in the grass AIT group versus 1 in the placebo group; all were assessed as mild in severity. No subjects in the grass AIT group discontinued due to asthma events. Two serious adverse events were reported, both related to falls and both in the placebo group.

Three subjects, all in the grass AIT group, were administered epinephrine. The first subject experienced a moderate anaphylactic reaction, as assessed by the investigator, 5 minutes after first dose. Symptoms included swelling of lips; itchy mouth, tongue, and throat; and dysphagia. The subject was treated on site with antihistamine and epinephrine; the event resolved, and the subject was withdrawn from the study. The second subject experienced itchy throat, itchy mouth, dry cough, labial hive, post-nasal drip, and uvula erythema immediately after first dose. The subject was treated on site with antihistamine, epinephrine, and oral prednisone. The event resolved, and the subject was withdrawn from the study. The third subject experienced a systemic allergic reaction assessed as mild by the investigator 6 minutes after first dose. Symptoms included itching under the tongue, throat, ears and nose, sneezing, rhinorrhea, and throat irritation. The subject was treated on site with antihistamine and epinephrine, and the event resolved. This subject experienced another systemic allergic reaction the next day which resolved without treatment, and the subject continued in the trial. Two additional subjects in the grass AIT group experienced systemic reactions (one subject: symptoms of lightheadedness, headache, sleepiness, and itching of ears; one subject: symptoms of lightheadedness and itching in mouth) after the first treatment dose but did not receive epinephrine. These subjects also recovered without sequelae and continued in the trial. There were no signs of hypotension in any of these 5 subjects.

## Discussion

In the current study, no significant differences between grass AIT and placebo were observed for the primary end point (mean AR/C DSS over the entire GPS) or for the key secondary end points, although trends in favor of AIT were seen. The design of the current study was modeled after a phase 3, placebo-controlled trial of 634 adults with grass pollen-induced AR/C that was conducted in 8 European countries [8]. In that study, grass AIT initiated an average of 26 weeks prior to the start of the GPS significantly reduced AR/C symptoms and medication use. The efficacy of grass AIT has been demonstrated in 4 phase 2 and 3 trials conducted in European subjects with AR/C due to grass pollen allergy [8-11]; 2 large, placebo-controlled phase 3 studies in North American subjects assessed symptoms during the 2009 GPS and found that pre- and co-seasonal treatment with grass AIT was well tolerated and significantly reduced AR/C symptoms and combined symptom and medication score [12,13]. As in other trials of grass AIT [8-13], there were no cases of anaphylactic shock in the present study; epinephrine was administered to 3 subjects, all of whom experienced symptoms within 10 minutes after the first dose of grass AIT and none of whom showed signs of hypotension.

Post hoc analyses of the present trial were attempted, to clarify what characteristics of its population or design might have contributed to its failure to achieve the results seen in successful trials of grass AIT. As in “real-world” conditions, many subjects in the study were sensitized to multiple allergens; approximately 50% of subjects were sensitized to tree pollen, cat hair, and HDM. However, subgroup analysis of subjects without these additional sensitizations also failed to show significant differences between grass AIT and placebo. Further, the proportion of subjects also sensitized to other common allergens in the present trial was similar to those seen in other trials of grass AIT, in which significant treatment effects were seen [8-13]. A recent pooled analysis of data from 6 such placebo-controlled randomized trials that found that response to grass AIT treatment was similar between subjects sensitized only to grass and subjects sensitized to other common allergens [14]. Given these pieces of evidence, it is considered unlikely that the efficacy of grass AIT was masked by the allergic responses to other common seasonal and perennial allergens.

However, several lines of evidence give support to the idea that the symptoms reported by subjects in this trial may not have been reflective of the influence of grass pollen exposure. First, the pattern of symptoms during the GPS was unexpected. In the current study, symptom severity and medication use did not show any clear relationship to seasonal pollen exposure. By comparison, symptoms generally mirrored pollen levels in other grass AIT studies [8,9,12,13], ie, peak symptoms coincided with peak pollen levels. Additionally, subjects in the current trial showed high pre-seasonal symptoms, whereas in other grass AIT trials symptoms were relatively minimal in the pre-seasonal period [12,13]. A post hoc analysis divided subjects into those with low pre-seasonal symptoms (DSS ≤3; 33% of all subjects) and those with high pre-seasonal symptoms (DSS >3; 67% of all subjects). In those with pre-seasonal DSS ≤3, mean DSS and mean DMS were both significantly lower over the GPS in the grass AIT group compared with the placebo group (27%; p = 0.0327 and 68%; p = 0.0060, respectively). The 27% reduction in symptom scores was similar to the magnitude of the treatment effect seen in other grass AIT trials (18% to 30% reduction in mean DSS relative to placebo), [8,12] and as in these trials the symptom scores closely corresponded to pollen exposure. In subjects with pre-season DSS >3, no significant differences between grass AIT and placebo were seen for mean DSS or DMS (p >0.05). Along with the fact that no relationship of symptoms to pollen count was observed in the overall population, this suggests that the symptoms reported in this trial were not primarily reflective of the effects of grass pollen exposure. It is conceivable that subjects were suffering symptoms due to some other unidentified cause, or that some subjects understood poorly the standards by which they were to score their symptoms.

## Conclusions

In this randomized, double-blind, placebo-controlled, multicenter North American trial, grass AIT at a dose of 2800 BAU was not associated with significant improvements in AR/C symptom or medication scores versus placebo over the GPS. These results contrast with the efficacy repeatedly demonstrated in European and 2 other North American trials of grass AIT for the treatment of AR/C. Multiple possibilities have been explored as plausible explanations for the trial failure. Though no firm conclusions can be made, the high pre-seasonal symptoms, lack of a relationship between pollen count and symptom score in the presence of significant immunological response, and the significant results of post hoc analysis excluding subjects with high pre-seasonal scores suggest that the symptoms reported were not primarily reflective of the effects of grass pollen exposure.

## Methods

### Study design

This was a phase 3, double-blind, randomized, placebo-controlled, parallel-group multicenter trial conducted at 28 sites in the United States (GT-14; clinicaltrials.gov identifier NCT00421655). The study was conducted in compliance with Good Clinical Practice guidelines. The protocol was approved by institutional review boards for each center. All subjects provided written informed consent before any study activity began.

### Treatment

Qualified subjects were randomized 1:1 to once-daily 2800 BAU of standardized Timothy grass AIT treatment (oral lyophilisate, *Phleum pratense*, 75,000 standardized quality tablet, containing approximately 15 μg of Phl p 5; ALK, Hørsholm, Denmark) or placebo (identical in composition, appearance, smell, and taste to active treatment but with no grass pollen extract included) with no build-up dosing. The tablets were supplied as fast-dissolving, neutral-tasting oral lyophilisates for sublingual application. Excipients included gelatin, mannitol, and sodium hydroxide. Treatment was administered sublingually, preferably in the morning, for at least 8 to 16 weeks before the anticipated start of the GPS and continuing throughout the GPS. Randomization was performed by in blocks by ALK, using the SAS^®^ system for Windows, which generates random assignment of treatment groups to randomization numbers. The randomization list was generated by a trial statistician who was independent of the statistical analyses. A 5-digit subject number was allocated to the subject at the screening visit (visit 1). When a subject was randomized in the trial he/she was always to be assigned the lowest available randomization number. The randomization number was a 4-digit number.

The first dose of study medication was administered at the study site. Subjects were required to remain at the study site for 20 to 30 minutes after administration of the first dose to monitor for any AEs. Subsequent treatments were self-administered by the subject once daily at home. Eating and drinking were not allowed for 5 minutes after administration. If any significant adverse event such as wheezing, dyspnea, severe oral swelling, or sign of generalized anaphylactic reaction was observed or reported, the investigator was to evaluate the subject to determine whether treatment should be initiated; in such cases the observation period was to be extended for at least an additional 30 minutes, and upon leaving the clinic the subject was instructed to contact the clinic immediately if the reaction reoccurred or a new reaction appeared.

### Study subjects

Subjects included in the study were 18 to 65 years of age with a clinical history of grass pollen-induced AR/C, with or without asthma, that interfered with daily activities or sleep and was bothersome despite symptomatic treatment during the GPS. At screening, subjects were required to meet the following criteria: positive skin prick test response to *Phleum pratense* defined as a wheal diameter ≥5 mm larger than that elicited by the saline control (standardized Timothy grass extract 100,000 BAU/mL, 5 mL [ALK, Hørsholm, Denmark] administered to the inner forearm with a DuoTip [Lincoln Diagnostics, Decatur, Ill]; positive control, histamine dihydrochloride 10 mg/mL [ALK, Hørsholm, Denmark]); positive specific IgE against *P pratense* (≥IgE Class 2 [≥0.7 kU/L; measured using the DPC Immulite 2000, Siemens Medical Solutions Diagnostics, Erlangen, Germany]); and an FEV_1_ of 70% or greater of predicted value. Key exclusion criteria were as follows: history of AR/C and/or asthma due to another allergen potentially overlapping GPS; history of significant symptomatic perennial or allergic rhinitis/asthma to an allergen to which the subject was regularly exposed; immunotherapy treatment within the previous 5 years; clinical history of severe asthma, angioedema, or chronic/recurrent rhinosinusitis or of chronic urticaria within the last year; or history of anaphylaxis.

Although Bermuda grass sensitivity was not specifically assessed (to exclude subjects with this allergy), only 3 of 28 study sites were located in the southern regions in which Bermuda grass is known to pollinate.

### Grass pollen season

One unique pollen count station recorded grass pollen counts at each site. The start of the GPS for each site was defined as the first 3 consecutive days with a pollen count of 10 grains/m^3^/day or greater, and the end of the GPS for each site was defined as the last day of the last occurrence of 3 consecutive days with a pollen count of 10 grains/m^3^/day or greater. The peak of the GPS was defined as the period of 15 consecutive recorded days with the highest average among all possible 15 consecutive-day averages across the GPS.

### Assessments

The primary end point of the study was the average AR/C symptom score during the entire GPS, calculated for each subject as the sum of the individual AR/C DSS divided by number of DSS diary recordings.

Key secondary end points were the average weekly score on the RQLQ(S) over the entire GPS; the average DMS, calculated as the sum of each day’s DMS divided by the number of DMS diary recordings during entire GPS; and the percentage of AR/C well days (days without any AR/C rescue medication and a DSS of ≤2) during the entire GPS.

The AR/C and asthma symptom scores were recorded once daily in an electronic diary from the pre-seasonal visit through the end of the GPS. The AR/C DSS was composed of 6 symptoms (runny nose, blocked nose, sneezing, itchy nose, gritty feeling/red/itchy eyes, and watery eyes) and the asthma DSS was composed of 4 symptoms (cough, wheeze, chest tightness/shortness of breath, and exercise-induced symptoms). All symptoms were measured as follows: 0, no symptoms; 1, mild symptoms; 2, moderate symptoms; or 3, severe symptoms. Open-label AR/C rescue medication was provided approximately 2 weeks before the start of the GPS to be used in a stepwise sequence once the start of the GPS had been confirmed and subjects reported a total AR/C symptom score ≥6 (Table 6). Asthma medication (for subjects with asthma) was provided as needed and use was recorded; the asthma DMS was composed of the sum of scores for short-acting β antagonist and inhaled corticosteroid use. Subjects were instructed to record their use of rescue medications in the electronic diary. The AR/C DMS was composed of the sum of scores for oral antihistamine and ocular antihistamine use. No other AR/C or asthma medications were allowed (ie, parenteral, oral, nasal, and inhaled corticosteroids, leukotriene antagonists, cromones, decongestants, long-acting β_2_-agonists, or additional topical or oral antihistamines). The RQLQ(S) was completed at visit 4 and weekly during the GPS. A higher score indicates more significant impairment.

**Table 6:** Schedule for rescue medication

**Step**	**Rescue medication**	**Score/dose unit**	**Maximum daily score**
Rhinoconjunctivitis			
1	Desloratadine tablets, 5 mg, 1 tablet QD	6 (per tablet)	6
2	Olopatadine eye drops, 1 mg/ml, 1 drop in the affected eye BID	1.5 (per drop)	6
Maximum daily rhinoconjunctivitis medication score			12
Asthma			
A	Albuterol inhalation powder, 120 μg/dose, up to 2–4 inhalations twice daily	1 (per 1 inhalation)	8
B	Fluticasone inhalation powder, 250 μg/dose, 1–2 inhalations up to twice daily	2 (per inhalation)	8
Maximum daily asthma medication score			16

Additional efficacy end points included AR/C DSS over the peak GPS, nose and eye symptoms over the peak and entire GPS, AR/C DMS over the peak GPS, combined AR/C DSS and DMS over the peak and entire GPS, AR/C DSS and DMS over the first 7 days of GPS, AR/C symptoms by visual analogue scale (VAS) score over peak and entire GPS, global evaluation of individual AR/C symptoms and overall global evaluation, excellent AR/C control (>50% well days), AR/C well days over peak GPS, days without AR/C rescue medication use or symptoms, asthma DSS over peak and entire GPS, asthma DMS over peak and entire GPS, and asthma well days (days without asthma rescue medication, with asthma DSS ≤1) over peak and entire GPS.

### Immunologic assessments

The serum was analyzed by ALK for determination of antigen-specific antibodies and other immunologic parameters (IgE, IgG4, and IgE-blocking antibodies).

Immunologic tests were performed on blood collected at the screening visit (visit 1), the pre-season visit (visit 4), and at the end-of-season visit (visit 6). Blood samples were analyzed by ALK by means of the ADVIA Centaur Immunoassay system (Siemens Medical Solutions Diagnostics, Tarrytown, NY).

### Safety

Safety was assessed through AEs that were spontaneously reported by subjects or observed by the investigator; at each site visit, investigators also asked the subjects whether any problems had occurred since the previous contact. AEs were graded by the investigators as mild (transient symptoms, no interference with the subject’s daily activities), moderate (marked symptoms, moderate interference with the subject’s daily activities), or severe (considerable interference with the subject’s daily activities, unacceptable). Safety assessments also included hematology, blood chemistry and urinalysis testing, physical examination, vital signs, and FEV_1_.

### Statistical analysis

Sample size calculations were based on data from a previous grass AIT trial [8] in which the mean values and standard deviations (SD) for the symptom score were 2.4 (1.6) for grass AIT and 3.4 (2.2) for placebo. Approximately 150 subjects per group, assuming a 20% dropout rate, would detect a 24% reduction in mean DSS for grass AIT compared with placebo at a 5% significance level and with 90% power. The difference between the grass AIT and placebo groups for DSS, DMS, and percentage of well days was assessed by analysis of variance (ANOVA), with treatment group as fixed effect and pollen region as random effect. The confidence interval for the relative treatment difference was estimated by bootstrapping using the mean estimates. The weekly overall RQLQ(S) analysis was performed using a repeated measurement ANOVA including treatment group, week, and treatment-by-week interaction as fixed effects, pollen area as a random effect, and adjusting for subject variation. An AR(1) or compound symmetry covariance structure was applied. All efficacy analyses were conducted on the intent-to-treat population based on all randomized subjects who had data available (at least 1 post-treatment diary data entry during the entire GPS, or during peak GPS for end points assessing peak GPS) for analysis. There was no imputation of missing data, and subjects who withdrew prior to the start of the GPS did not contribute to the efficacy analyses. Safety analyses were conducted on all randomized subjects. Differences in the immunological assessments between visits within treatment and differences between treatments at each visit were estimated using a repeated measurement model. The response variable in the model was change from baseline. Treatment, visit, treatment by visit interaction, and pollen area were included as fixed effects, and the adjustment for different error variation for each group was performed. IgE and IgG4 values were log10-transformed to obtain approximately normally distributed data. Differences between visits in immunological measurements within each treatment were in addition tested using a Student’s *t*-test; non-parametric tests of differences between treatments were performed using a Wilcoxon test. The principal statistical software used was SAS^®^, version 8.2.

## Abbreviations

AEs: Adverse events; AIT: Allergy immunotherapy tablet; ANOVA: Analysis of variance; AR(1): Autoregressive covariance model (1); AR/C: Allergic rhinitis with or without conjunctivitis; BAU: Bioequivalent allergen units; DMS: Daily medication score; DSS: daily symptom score; FEV1: Forced expiratory volume in 1 second; GPS: Grass pollen season; HDM: House dust mite; RQLQ(S): Rhinoconjunctivitis quality of life questionnaire with standardized activities; SD: Standard deviations; VAS: Visual analogue scale.

## Competing interests

Kevin Murphy reports advisory board membership, speaker's bureau participation, and consultancy for Merck. Sandra Gawchik reports clinical research support, consultancy, travel support, and ad board participation for Merck, and speaker’s bureau income from AstraZeneca. David Bernstein reports consultant fees/honoraria from Merck and ALK-Abelló, travel support from Merck, fees for review activities from Merck and ALK-Abelló, consultancy for Proctor & Gamble and Sanofi-Aventis, expert testimony for law firms, and grants/grants pending with NIOSH (CDC), NIAID, and NIEHS. Jens Andersen and Martin Rud Pedersen are employees of ALK-Abelló, the study sponsor.

## Authors’ contributions

KM, SG, and DB enrolled study subjects and carried out the study protocol. JS and MRP conceived the study, and participated in its design and coordination; JS contributed to the statistical analysis. All authors helped to draft the manuscript and read and approved the final manuscript.

## Authors’ information

KM, SG, and DB are United States-based physicians and researchers with interests relating to allergic rhinitis with or without conjunctivitis. JS and MRP are employees of ALK-Abelló and contribute to the clinical development of sublingual immunotherapy tablets, a therapeutic modality common in Europe but considered investigational in the United States.

## Trial support

This study was funded by ALK-Abelló, Hørsholm, Denmark.
